# Genome-Wide Association for Itraconazole Sensitivity in Non-resistant Clinical Isolates of *Aspergillus fumigatus*

**DOI:** 10.3389/ffunb.2020.617338

**Published:** 2021-01-14

**Authors:** Shu Zhao, Wenbo Ge, Akira Watanabe, Jarrod R. Fortwendel, John G. Gibbons

**Affiliations:** ^1^Molecular and Cellular Biology Graduate Program, University of Massachusetts, Amherst, MA, United States; ^2^Department of Food Science, University of Massachusetts, Amherst, MA, United States; ^3^Department of Clinical Pharmacy and Translational Science, University of Tennessee Health Science Center, Memphis, TN, United States; ^4^Division of Clinical Research, Medical Mycology Research Center, Chiba University, Chiba, Japan; ^5^Organismic and Evolutionary Biology Graduate Program, University of Massachusetts, Amherst, MA, United States

**Keywords:** mycology, fungal pathogen, genome-wide association, population genomics, azole, antifungal, *Aspergillus fumigatus*, itraconazole

## Abstract

*Aspergillus fumigatus* is a potentially lethal opportunistic pathogen that infects over ~200,000 people and causes ~100,000 deaths per year globally. Treating *A. fumigatus* infections is particularly challenging because of the recent emergence of azole-resistance. The majority of studies focusing on the molecular mechanisms underlying azole resistance have examined azole-resistant isolates. However, isolates that are susceptible to azoles also display variation in their sensitivity, presenting a unique opportunity to identify genes contributing to azole sensitivity. Here, we used genome-wide association (GWA) analysis to identify loci involved in azole sensitivity by analyzing the association between 68,853 SNPs and itraconazole (ITCZ) minimum inhibitory concentration (MIC) in 76 clinical isolates of *A. fumigatus* from Japan. Population structure analysis suggests the presence of four distinct populations, with ITCZ MICs distributed relatively evenly across populations. We independently conducted GWA when treating ITCZ MIC as a quantitative trait and a binary trait, and identified two SNPs with strong associations in both analyses. These SNPs fell within the coding regions of *Afu2g02220* and *Afu2g02140*. We functionally validated *Afu2g02220* by knocking it out using a CRISPR/Cas9 approach, because orthologs of this gene are involved in sterol modification and ITCZ targets the ergosterol biosynthesis pathway. Knockout strains displayed no difference in growth compared to the parent strain in minimal media, yet a minor but consistent inhibition of growth in the presence of 0.15 μg/ml ITCZ. Our results suggest that GWA paired with efficient gene deletion is a powerful and unbiased strategy for identifying the genetic basis of complex traits in *A. fumigatus*.

## Introduction

Fungal infections result in more global deaths per year than deaths from tuberculosis or malaria (Brown et al., 2012). *Aspergillus fumigatus* is one of the most deadly fungal pathogens and results in more than 100,000 deaths per year (Brown et al., 2012). Invasive aspergillosis (IA) is the most severe infection caused by *A. fumigatus* and occurs when fungal growth, most commonly originating in the lung, disseminates to other parts of the body via the bloodstream (Latge, 1999). *A. fumigatus* is an opportunistic pathogen primarily affecting immunocompromised individuals, and unfortunately, infections have become more common due to the increased usage of immunosuppressive drugs to treat autoimmune disorders and to increase the success of organ transplantation surgery (Robinett et al., 2013; Neofytos et al., 2018; Latge and Chamilos, 2019). Even when aggressively treated with first and second-line antifungal medication, mortality rates can exceed 50% in IA patients (Latge, 1999; Lin et al., 2001). The relatively rapid emergence of *A. fumigatus* antifungal resistance has made treatment of infections particularly challenging.

Antifungal drugs target components that distinguish fungal cells from mammalian cells, including the fungal cell wall as well as unique components of the fungal cell membrane. For example, the echinocandins target β 1,3 glucan, the most abundant polysaccharide in the fungal cell wall, while amphotericin B (a polyene class of antifungal drug) and triazoles (an azole class of antifungal drugs) target ergosterol (Latge et al., 2017). Ergosterol plays an essential functional role in regulating cell membrane permeability and fluidity. Triazoles, such as itraconazole (ITCZ) and voriconazole, are the most common first-line treatment for *A. fumigatus* infections, and target the lanosterol demethylase enzymes (Cyp51A and Cyp51B in *A. fumigatus*) which are directly involved in the biosynthesis of ergosterol (Alcazar-Fuoli and Mellado, 2013; Revie et al., 2018). Blocking Cyp51A and Cyp51B results in the accumulation of a toxic sterol intermediate that causes severe membrane stress, impairment of growth, and cell death (Revie et al., 2018).

Strains of *A. fumigatus* have gained resistance to triazoles through mutations in both the coding and regulatory regions of *cyp51A*, and through *cyp51A* independent mechanisms (Garcia-Rubio et al., 2017). The three amino acid positions that are commonly found with non-synonymous mutations in *cyp51A* in azole resistance strains are 54, 220, and 448 (Garcia-Rubio et al., 2017). Protein structure modeling suggests that these mutations disrupt the binding efficiency of azoles to Cyp51A (Fraczek et al., 2011; Warrilow et al., 2015). Increased expression of *cyp51A* through a combination of a promoter region repeat and the L98H point mutation can also confer azole resistance (Mellado et al., 2007). Additionally, several transcription factors (e.g., *srbA* (Hagiwara et al., 2016), *hapE* (Camps et al., 2012), *atrR* (Paul et al., 2019), transporters [e.g., *cdr1B* (Fraczek et al., 2013), *atrF* (Meneau et al., 2016), various ABC transporters (Moye-Rowley, 2015) etc.], and other functional groups of genes (e.g., genes involved in calcium signaling, iron balance, signaling pathways, and the Hsp90-calcineurin pathway) have been implicated in azole resistance or susceptibility (Chen et al., 2020).

The numerous genes identified in azole resistance other than *cyp51A* (Garcia-Rubio et al., 2017) suggests that additional genes with additive minor effects likely play a role in fine-scale differences in azole sensitivity and resistance. Historically, most genes involved in azole resistance in *A. fumigatus* were discovered through a candidate gene approach (Garcia-Rubio et al., 2017), or through gene expression differences during exposure to azoles (da Silva Ferreira et al., 2006; Hokken et al., 2019). However, candidate gene methods are biased toward genes and pathways of biological interest. Alternatively, genome-wide association (GWA) studies offer a powerful and versatile approach to identify genetic variants that contribute to complex traits, such as *A. fumigatus* ITCZ sensitivity. In GWA, thousands to millions of high-density genetic variants are tested for a statistical association between each variant and a phenotype of interest (Gibson, 2018). Microbial GWAS methods have recently been developed (Read and Massey, 2014; Chen and Shapiro, 2015; Power et al., 2017), and has been used in other fungal species. For instance, GWA has been used to identify genes and variants associated with virulence in *Heterobasidion annosum* (Dalman et al., 2013), *Saccharomyces cerevisiae* (Muller et al., 2011), and *Parastagonospora nodorum* (Gao et al., 2016), fungal communication in *Neurospora crassa* (Palma-Guerrero et al., 2013), aggressiveness in *Fusarium graminearum* (Talas et al., 2016), and *Zymoseptoria tritici* (Hartmann et al., 2017). Here, we hypothesized that GWA could be applied in *A. fumigatus* to identify genes with minor effects on ITCZ sensitivity. We performed GWA in 76 non-resistant clinical isolates of *A. fumigatus* and identified a gene that contributes to fine-scale ITCZ sensitivity. More broadly, we demonstrate that GWA in combination with gene disruption is a useful tool for investigating medically relevant traits in *A. fumigatus*.

## Materials and Methods

### Japanese *A. fumigatus* Clinical Isolates

Sixty-five Japanese *A. fumigatus* clinical strains were provided through the National Bio-Resource Project (NBRP), Japan (http://nbrp.jp/) (Supplementary Table 1). These samples originated from different patients, several different sources and infections, and 15 cities. Whole genome paired-end Illumina sequence data for an additional 11 *A. fumigatus* isolates that were previously sequenced and have ITCZ MIC data (Takahashi-Nakaguchi et al., 2015) (Supplementary Table 1) were downloaded from NCBI Sequence Read Archive (SRA) (Leinonen et al., 2011) using the SRA toolkit (https://trace.ncbi.nlm.nih.gov/Traces/sra/sra.cgi?cmd=show&f=software&m=software&s=software).

### Minimum Inhibitory Concentration Testing

Minimal inhibitory concentration (MIC) of ITCZ for each isolate was determined following the Clinical and Laboratory Standards Institute (CLSI) M38-A2 broth microdilution method (John, 2008). Before MIC calculations, each strain was cultured using a potato dextrose agar plate (Becton Dickinson, Sparks, MD, US) for 5 days at 30°C degrees to produce the fungal conidia. Harvested conidia were suspended in standard RPMI 1640 broth (pH = 7) (Sigma Aldrich, St. Louis, US-MO). For each isolate, 2.5 × 10^4^ conidia per l mL were incubated in RPMI 1640 broth (pH=7) with a range of ITCZ concentrations (8, 4, 2, 1, 0.5, 0.25, 0.125, 0.0625, 0.03125, 0.015625 μg/ml) at 35°C for 48 h. MIC values represent the lowest ITCZ concentrations that completely inhibited growth.

### DNA Extraction and Illumina Whole-Genome Sequencing

Genomic DNA (gDNA) isolation was performed as previously described (Zhao et al., 2019). gDNA was directly isolated from conidia stocks using the MasterPure™ Yeast DNA Purification Kit (Lucigen/Epicenter) following the manufacturer's instructions, with several minor modifications. Conidia stocks were centrifuged at 14,000 RPM for 5 min to obtain a pellet. Next, 300 ml of yeast cell lysis solution was added to the pellet along with 0.4 ml of sterile 1.0 mm diameter silica beads. Lysis was carried out on a Biospec Mini-BeadBeater-8 at medium intensity for 8 min. One μl of RNase was added to the cell lysis solution and incubated at 65°C for 30 min. DNA isolation and purification were conducted according to the manufacturer's instructions for the remainder of the protocol. PCR-free 150-bp paired-end libraries were constructed and sequenced by Novogene (https://en.novogene.com/) on an Illumina NovaSeq 6000.

### Quality Control and Sequence Read Mapping

Raw reads were first deduplicated using tally (Davis et al., 2013) with the parameters “–with-quality” and “–pair-by-offset” to remove potential PCR duplication during library construction. Next, we used trim_galore v0.4.2 (http://www.bioinformatics.babraham.ac.uk/projects/trim_galore/) to trim residual adapter sequences from reads, and trim reads where quality score was below 30, with the parameters “–stringency 5” and “-q 30,” respectively. Trimmed reads shorter than 50 bp were then discarded using the option “–length 50.” Next, the deduplicated and trimmed read set was mapped to the *A fumigatus* Af293 reference genome (Nierman et al., 2005) using BWA-MEM v0.7.15 aligner (Li and Durbin, 2009). The resulting SAM files were converted into sorted BAM files using the “view” and “sort” functions in samtools 1.4.1 (Li et al., 2009).

### SNP Genotyping

Because *A. fumigatus* is haploid, we followed the best practice pipeline for “Germline short variant discovery” (Van Der Auwera et al., 2013) in Genome Analysis ToolKit (GATK) v4.0.6.0 (Mckenna et al., 2010). The function “HaplotypeCaller” was used to call short variants (SNPs and INDELs) with the sorted BAM file for each sample. The resulting g.vcf files of all 76 samples were then combined to generate a joint-called variant file using the function “GenotypeGVCFs.” Next only SNPs were extracted from the joint-called variant file using the function “SelectVariants.” To limit false positive variant calling, the function “VariantFiltration” was used to carry out “hard filtering” with the following parameters: “QD < 25.0 || FS > 5.0 || MQ < 55.0 || MQRankSum < −0.5 || ReadPosRankSum < −2.0 || SOR > 2.5”. 206,055 polymorphic loci were predicted after hard filtering.

### Population Structure of *A. fumigatus* Isolates

To investigate the population structure of the *A. fumigatus* isolates we used a subset of population genetic informative SNPs. We used VCFtools v0.1.14 (Danecek et al., 2011) (http://vcftools.sourceforge.net/) with options “–maf 0.05 –max-missing 1 –thin 3500,” to filter the full set of SNPs and require a minor allele frequency ≥ 5%, no missing data across all samples, and at least 3.5 Kb distance between SNPs. 6,324 SNPs remained after filtering, and subsequent population structure analysis was conducted with this marker set. In addition, to test the consistency of population assignments with different number of SNPs, population structure analysis was conducted with a dense SNP set where thinning was not applied (59,433 SNP sites) and an additional thinned SNP set where markers were spaced apart by at least 35 Kb (756 SNPs).

To conduct population structure analysis, we first used the model-based program ADMIXTURE v1.3 (Alexander et al., 2009) for *K* = 1–10, where *K* indicates the number of populations. The 5-fold cross-validation (CV) procedure was calculated to find the most likely *K* with option “–cv = 5.” For each *K* the CV error was calculated and the *K* with lowest CV error indicated the most likely population number. Additionally, we used the non-model based population structure software DAPC (Jombart et al., 2010) in the “adegenet” package v2.1.2 (Jombart, 2008) in R v3.5.3 (Team, 2013) to the predict the number and assignment of individuals into populations. DAPC applies a Bayesian clustering method to identify populations without evolutionary models. The most likely number of populations was inferred by calculating the Bayesian Information Criterion (BIC) for each *K*.

Lastly, we also constructed a phylogenetic network with the alignment of 6,324 SNPs. The phylogenetic network was built using SplitsTree v4.14.4 (Huson and Bryant, 2006) with the neighbor joining method and 1,000 replicates for bootstrap analysis.

### Genome-Wide Association Analysis for Itraconazole Sensitivity

Genome Wide Association (GWA) analysis was conducted to identify genetic variants that were significantly correlated with ITCZ MIC. For GWA analysis, we filtered our complete set of SNPs with VCFtools to include SNPs with a minor allele frequency ≥5%, SNP sites with ≤ 10% missing data, and SNPs that were biallelic. This filtering procedure resulted in 68,853 SNPs that were used for GWA.

Two models were used to perform GWA between each of the 68,853 SNPs and ITCZ MIC. When ITCZ MIC data was treated as a quantitative trait (Supplementary Table 1), we used a linear mixed model with a genetic distance matrix for population structure correction in Tassel (Bradbury et al., 2007). GWA was also performed when ITCZ MIC was treated as a binary trait (MIC ≤ 0.5 = more sensitive, and MIC > 0.5 = less sensitive). In this GWA analysis, we used a mixed effect logistic model with an empirical covariance matrix as a population structure correction in RoadTrips (Thornton and Mcpeek, 2010). Quantile–quantile(Q-Q) plots were generated using the R package “qqman” (Turner, 2014) in order to evaluate potential *p*-value inflation. The potential functional effects of candidate SNPs were predicted using SnpEff v4.3t (Cingolani et al., 2012) with the *A. fumigatus* Af293 reference genome annotation.

### RNA-Seq Based Expression Data for *Afu2g02220* and *Afu2g02140*

To investigate the expression patterns of our candidate genes *Afu2g02220* and *Afu2g02140*, we obtained FPKM values as well as fold-change and *p*-values for pairwise comparisons from FungiDB (https://fungidb.org/fungidb/) (Stajich et al., 2012) for oxidative stress, iron depletion, growth in blood and minimal media, and ITCZ exposure (Irmer et al., 2015; Kurucz et al., 2018).

### Gene Deletion of *Afu2g02220* in *A. fumigatus* CEA10

*A. fumigatus* strain CEA10 was used as the genetic background for the deletion of *Afu2g02220*. The deletion was carried out using a clustered regularly interspaced short palindromic repeats (CRISPR)/Cas9-mediated protocol for gene editing, as previously described (Al Abdallah et al., 2017). Briefly, two Protospacer Adjacent Motif (PAM) sites, at both upstream and downstream of *Afu2g02220*, were selected using the EuPaGDT tool (Peng and Tarleton, 2015) and custom crRNAs were designed using the 20 base pairs of sequence immediately upstream of the PAM site. The crRNAs used are as follows: 5′ crRNA of *Afu2g0222*0 = CTGTTATTTTCTTCGGGTCT and 3′ crRNA of *Afu2g02220* = TGGACCAGGAAGAAACTGAG. Both crRNAs were purchased from IDT (Integrated DNA Technologies, Inc.). Complete guideRNAs (gRNAs) were then assembled *in vitro* using the custom designed crRNA coupled with a commercially acquired tracrRNA. The assembled gRNAs were then combined with commercially purchased Cas9 to form ribonucleoproteins for transformation, as previously described (Al Abdallah et al., 2017). Repair templates carrying a hygromycin resistance (HygR) cassette were PCR amplified to contain 40-basepair regions of microhomology on either side for homologous integration at the double strand DNA break induced by the Cas9 nuclease. Protoplast-mediated transformations were then carried out using the hygromycin repair templates and Cas-ribonucleoproteins for gene targeting. Homologous integrations were confirmed by PCR. The primers used are as follows:

*Afu2g02020* KO Forward Screening Primer (P1): GGATGCGTTGTTCCTGTGCG
*Afu2g02220* KO Reverse Screening Primer (P2): AACGAGGGCTGGAGTGCC
Common *HygR* Reverse Screening Primer (P3): ACACCCAATACGCCGGCC

### Comparison of ITCZ Sensitivity Between WT and KO Strains

Colony diameter was used as an estimate of growth rate to compare KO and WT strains in the presence and absence of ITCZ. For each strain, 10^4^ conidia were inoculated onto glucose minimal media (GMM) agar plates without ITCZ or with 0.15 μg/ml ITCZ. We used 0.15 μg/ml ITCZ because the parent strain (CEA10) is sensitive to ITCZ and did not grow in ITCZ concentrations ≥ 0.30 μg/ml. GMM was prepared as previously described (Shimizu and Keller, 2001). Colony diameter was measured with a digital caliper after 72 h at 37°C. Experiments were performed with ten replicates. To compare the growth rate between WT and KO strains, an ANOVA was performed between WT, Δ*Afu2g02220*-1, and Δ*Afu2g02220*-2 followed by a *post-hoc* Dunnett's test using the WT as the control group. Statistical analysis was conducted using JMP®, PRO 14 (SAS Institute Inc., Cary, NC, 1989–2019).

## Results

### Population Structure of Clinical *A. fumigatus* Isolates From Japan

We conducted whole genome sequencing (WGS) for 65 isolates of *A. fumigatus* from Japan and analyzed them in combination with an additional 11 previously sequenced isolates (Takahashi-Nakaguchi et al., 2015). Deduplicated, quality trimmed, and adapter trimmed WGS data of the 76 isolates were used for joint SNP calling with GATK (Mckenna et al., 2010) and yielded 206,055 SNPs. To reduce the linkage between adjacent SNPs for population structure analysis, we subsampled SNPs so that they were separated by at least 3.5 kb, which yielded 6,324 SNPs. This subsampled dataset was used for population structure and phylogenetic analysis.

**Figure 1:**
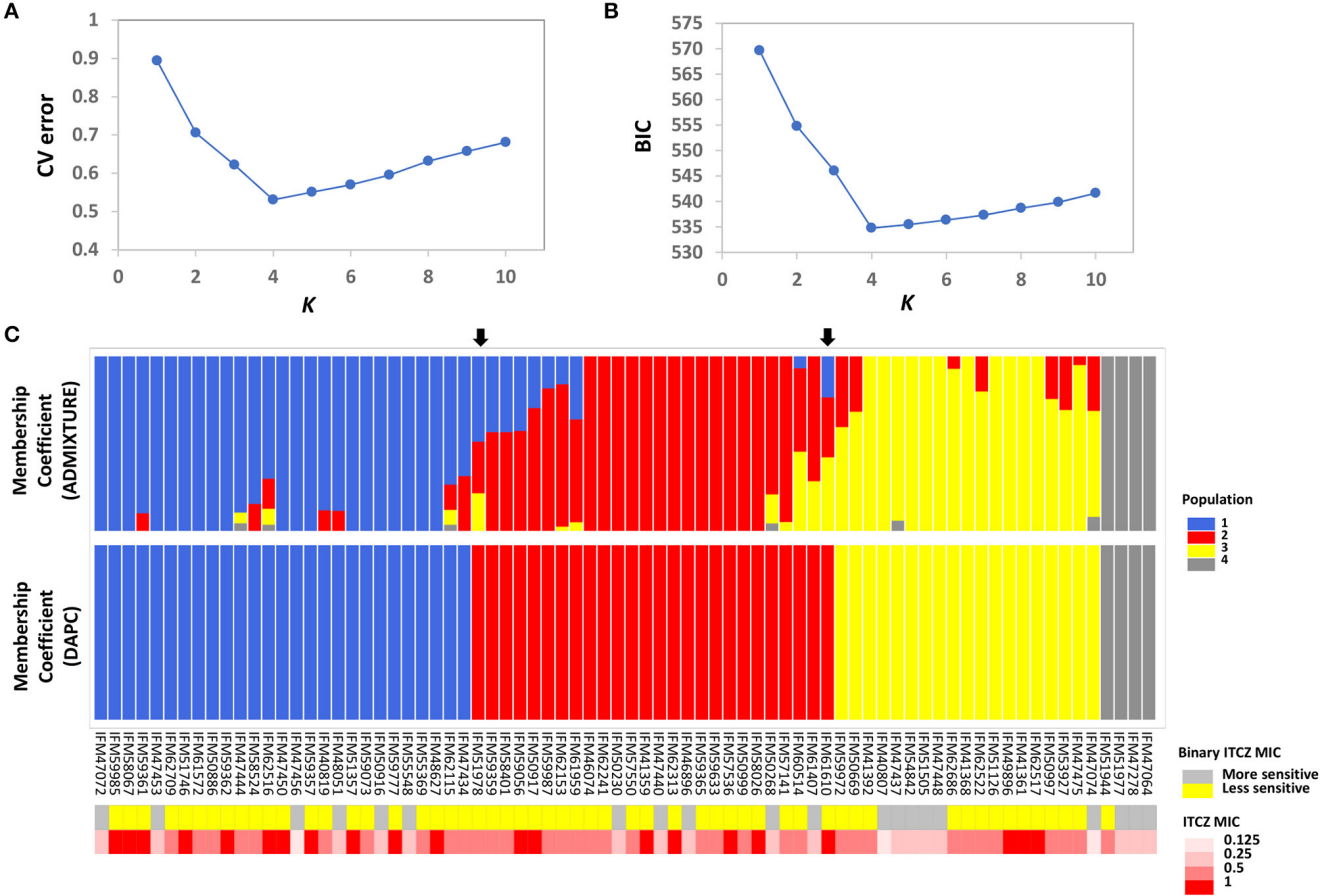
Population structure and ITCZ sensitivity of the 76 Japanese clinical *Aspergillus fumigatus* Isolates. **(A)** The optimal number of genetic clusters (*K*, X-axis) inferred by ADMIXTURE using cross validation procedure (CV error, Y-axis). **(B)** The optimal number of genetic clusters (*K*, X-axis) inferred by DAPC using Bayesian Information Criterion (BIC, Y-axis). **(C)** Membership coefficients (Y-axis) for each of the 76 isolates (X-axis) from ADMIXTURE and DAPC for *K* = 4. The two black arrows indicate the two isolates that are assigned into different clusters by ADMIXTURE and DAPC. Population 1, 2, 3, and 4 are colored as blue, red, yellow, and gray, respectively. Binary ITCZ MIC assignment and quantitative ITCZ MIC values are depicted in the upper and lower panels below the membership coefficient plots, respectively. For binary ITCZ MIC, individuals are coded as either more-sensitive (MIC < 0.5, gray) or less-sensitive (MIC ≥ 0.5, yellow).

Population structure is a main confounding factor in GWA studies that can lead to false positive associations (Sul et al., 2018). Therefore, we investigated the population structure of the 76 *A. fumigatus* isolates using the model-based approach implemented in ADMIXTURE (Alexander et al., 2009), as well as a non-model approach where population structure is inferred using discriminant analysis of principal components (DAPC) (Jombart et al., 2010). In ADMIXTURE, cross-validation (CV) error was estimated for each *K* from *K* = 1–10. The CV error is calculated by systematically withholding data points, and the lowest value represents the best estimate of the number of ancestral populations (Alexander and Lange, 2011). Using this approach *K* = 4 was the most likely population number (Figure 1A). DAPC uses the Bayesian Information Criterion (BIC) to evaluate the optimal number of clusters (*K*). *K* = 4 was also the most likely scenario as evaluated by BIC in DAPC (Figure 1B). Population assignment was highly consistent when the entire SNP set was used, or when subsampled datasets consisting of 6,324 or 756 markers were used to limit linkage between markers (Supplementary Figure 1). At *K* = 4, DAPC assigned the 76 isolates into four distinct populations with no admixture, while ADMIXTURE assigned 30 of 76 individuals to more than one population. For population assignment, we placed isolates into their respective population based on their largest membership coefficient. Using this approach, only two isolates, IFM51978 and IFM61610 (Figure 1C, indicated by black arrows), were assigned into different populations between the two methods. Phylogenetic network analysis further supports the presence of four main populations and individual population assignment into these populations (Supplementary Figure 2).

### Itraconazole Minimum Inhibitory Concentration

The ITCZ MIC of all isolates ranged from 0.125 to 1 μg/ml (MIC_0.125_ = 3, MIC_0.25_ = 17, MIC_0.50_ = 35, and MIC_1_ = 21). For reference, ITCZ resistance is typically defined by MIC ≥ 4 (Tashiro et al., 2012). GWA was independently conducted when MIC data was treated as a quantitative trait, and when MIC was treated as a binary trait (“more-sensitive” = MIC < 0.5 or “less-sensitive” = MIC ≥ 0.5). Populations 1, 2, 3, and 4 had 1, 0, 2, and 0 individuals with MIC = 0.125, 5, 5, 4, and 3 individuals with MIC = 0.25, 10, 14, 10, and 1 individuals with MIC = 0.5, and 11, 7, 3, and 0 individuals with MIC = 1, respectively (Figure 1C).

### Genome-Wide Association of Itraconazole Sensitivity in *A. fumigatus*

**Figure 2:**
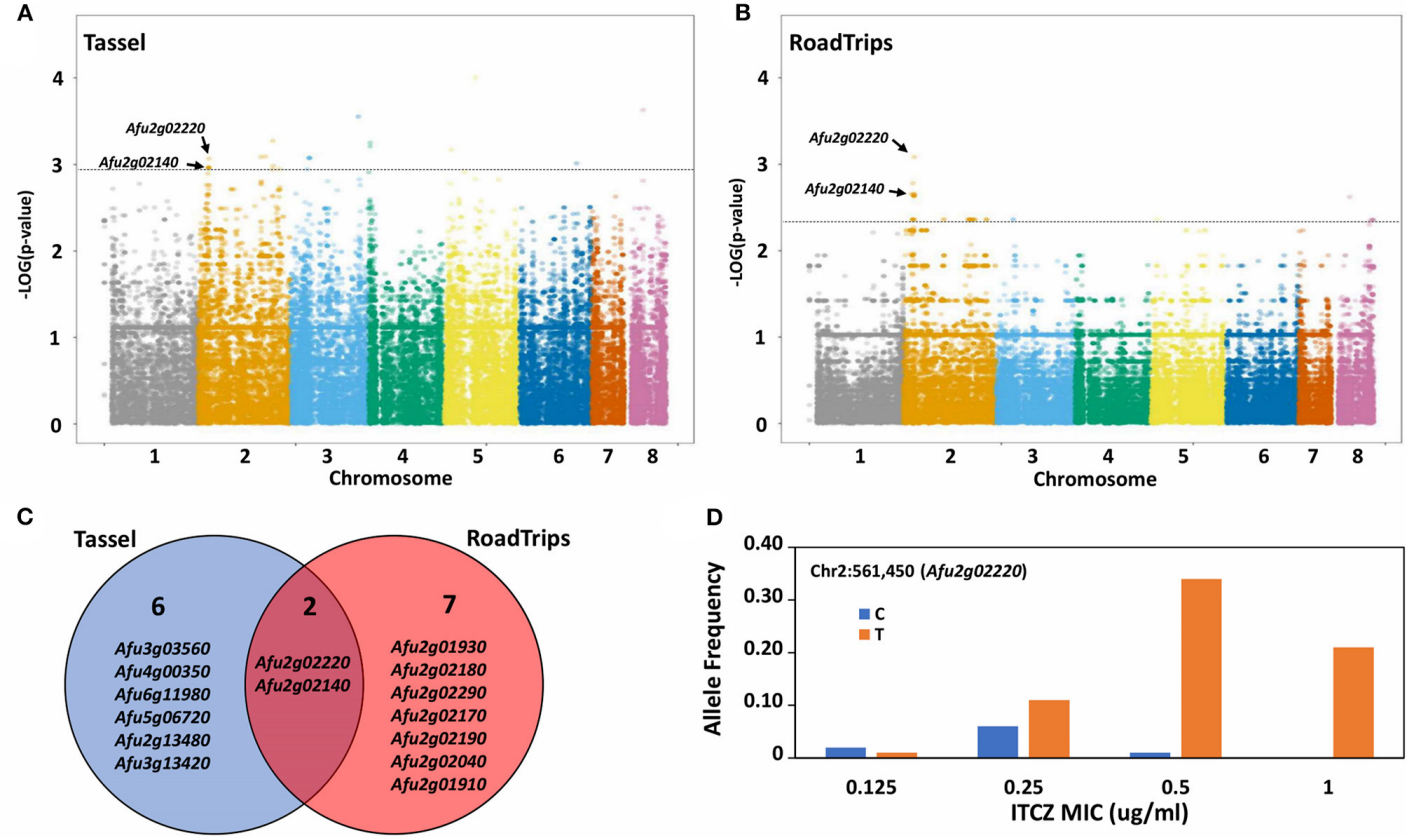
Genome-wide association (GWA) for itraconazole (ITCZ) sensitivity. GWA for ITCZ sensitivity when MIC data is treated as a quantitative trait **(A)** or as a binary trait **(B)**. For binary characterization of ITCZ sensitivity, MIC < 0.5 = more-sensitive and MIC ≥ 0.5 = less-sensitive. The genomic location of the 68,853 SNPs used for GWA is depicted on the X-axis, while the –log (*P-values*) are depicted on Y-axis. The dotted gray horizontal line represents the cutoff line at the 20th lowest *p-*value. *Afu2g02140* and *Afu2g02220* were within the 20 SNPs with the strongest associations in both analyses and are labeled on each plot. **(C)** Venn diagram of the 20 SNPs most strongly associated with ITCZ MIC that overlap genes when data is treated as a quantitative trait (blue circle) and a binary trait (red circle). **(D)** Allele frequency of the SNP at Chr2:561,450 that falls within *Afu2g02220* (Y-axis) by ITCZ MICs (X-axis).

We hypothesized that GWA would allow us to identify genes and/or genetic variants with minor contributions to ITCZ sensitivity. To test this hypothesis, we performed GWA with a set of 68,853 SNPs that have a minor allele frequency >5% and < 10% missing data, and the matched ITCZ MICs. Because these isolates have clear population structure (Figure 1) we used a mixed effect model GWA, which can reduce the inflated false-positive effect stemming from population structure (Yu et al., 2006; Price et al., 2010; Power et al., 2017) and has previously been applied in microbial GWA (Alam et al., 2014; Earle et al., 2016). We performed this mixed-model GWA with a covariance matrix as population correction for ITCZ MIC when treated as a quantitative trait (Figure 2A) and as a binary trait (Figure 2B) using Tassel 5 (Bradbury et al., 2007) and RoadTrips (Thornton and Mcpeek, 2010), respectively. We generated quantile-quantile (Q-Q) plots of expected vs. observed *p*-values to inspect *p*-value inflation, which could be the product of inadequate population structure correction. The Q-Q plots indicate that the distribution of *p*-values for both analyses are not inflated (Supplementary Figure 3).

We considered the 20 SNPs with the lowest *p*-values (lower 0.03 percentile) in each analysis as significant (Table 1). Of the 20 SNPs significantly associated with ITCZ MIC when MIC was treated as a quantitative trait (Figure 2A), five SNPs were located in genes (four in exons and one in an intron), 7 SNPs were located in 3′ UTR regions, two SNPs were located in 5′ UTR regions, and six SNPs were located in intergenic regions (Table 1). Of the four SNPs located in exons, one was synonymous (in *Afu2g02220*) while the remaining three SNPs were non-synonymous (in *Afu2g02140, Afu4g00350*, and *Afu6g11980*) (Table 1). Significant SNPs mapped to chromosomes 2 (*N* = 5), 3 (*N* = 8), 4 (*N* = 2), 5 (*N* = 2), 6 (*N* = 1), and 8 (*N* = 1) (Figure 2A).

**Table 1:** Characterization of SNPs associated with ITCZ sensitivity.

**Chr**.	**Pos**.	**Ref**	**Alt**.	**Tassel *p*-value**	**RoadTrips *p*-value**	**Gene ID**	**Predicted effect**
2	476106	G	T	0.00826	0.0043564^*^	*Afu2g01910*	Missense variant
2	478090	C	T	0.00826	0.0043564^*^	*Afu2g01910*	Synonymous variant
2	482083	T	C	0.00415	0.0016581^*^	*Afu2g01930*	Missense variant
2	483220	T	A	0.0028	0.00221361^*^	*Afu2g01930*	Missense variant
2	506066	C	G	0.00474	0.0043564^*^	*Afu2g02040*	Intron variant
2	534384	C	T	0.00173	0.00221361^*^	*Afu2g02140*	Synonymous variant
2	535564	G	C	0.00082113^*^	0.00231196^*^	*Afu2g02140*	Missense variant
2	536173	T	C	0.00109	0.00231196^*^	*Afu2g02140*	Missense variant
2	541570	T	C	0.00109	0.00231196^*^	*Afu2g02170*	5 prime UTR variant
2	543033	T	C	0.00109	0.00231196^*^	*Afu2g02170*	Synonymous variant
2	543252	G	A	0.00109	0.00231196^*^	*Afu2g02170*	Synonymous variant
2	549368	T	C	0.00109	0.00231196^*^	*Afu2g02180*	Upstream gene variant
2	550095	A	T	0.00109	0.00231196^*^	*Afu2g02190*	3 prime UTR variant
2	550165	T	G	0.00109	0.00231196^*^	*Afu2g02190*	3 prime UTR variant
2	561450	T	C	0.00081303^*^	0.000825977^*^	*Afu2g02220*	Synonymous variant
2	579284	C	T	0.00173	0.00221361^*^	*Afu2g02290*	Synonymous variant
2	3496859	C	A	0.00104^*^	0.00851735	*Afu2g13480*	3 prime UTR variant
3	953774	C	A	0.00028012^*^	0.108915	*Afu3g03560*	3 prime UTR variant
3	953900	T	C	0.00028012^*^	0.108915	*Afu3g03560*	3 prime UTR variant
3	953968	C	G	0.00084242^*^	0.108915	*Afu3g03560*	3 prime UTR variant
3	953975	G	C	0.00084242^*^	0.108915	*Afu3g03560*	3 prime UTR variant
3	954072	A	C	0.00084242^*^	0.108915	*Afu3g03560*	3 prime UTR variant
3	954106	T	C	0.00084242^*^	0.108915	*Afu3g03560*	3 prime UTR variant
3	3551729	A	T	0.00084242^*^	0.347887	*Afu3g13420*	5 prime UTR variant
3	3551730	A	T	0.00084242^*^	0.347887	*Afu3g13420*	5 prime UTR variant
4	95177	G	C	0.00061923^*^	0.354433	*Afu4g00350*	Missense variant
5	1649986	A	G	0.00067773^*^	0.221647	*Afu5g06720*	Intron variant
6	2995822	G	A	0.00097218^*^	0.221647	*Afu6g11980*	Missense variant
2	3271604	A	G	0.00053335^*^	0.153849	*Afu2g12760-Afu2g12770*	Intergenic region
2	3894996	G	C	0.00109^*^	0.120241	*Afu2g14770-Afu2g14780*	Intergenic region
2	3908952	A	C	0.0008576^*^	0.250408	*Afu2g14800-Afu2g14810*	Intergenic region
4	93588	G	C	0.00056303^*^	0.884311	*Afu4g00340-Afu4g00350*	Intergenic region
5	399165	A	T	0.000099305^*^	0.118454	*Afu5g01540-Afu5g01550*	Intergenic region
8	627169	G	C	0.00023513^*^	0.135283	*Afu8g02340-Afu8g02350*	Intergenic region
2	413387	G	A	0.00173	0.00221361^*^	*Afu2g01680-Afu2g01690*	Intergenic region
2	505989	C	T	0.00474	0.0043564^*^	*Afu2g02030-Afu2g02040*	Intergenic region
2	514248	T	C	0.00474	0.0043564^*^	*Afu2g02070-Afu2g02080*	Intergenic region
8	590672	C	T	0.02195	0.00238334^*^	*Afu8g02250-Afu8g02255*	Intergenic region

* *p-value is significant in the corresponding method*.

*Chr., chromosome; Pos., position; Ref., reference allele; Alt., Alternate allele*.

Of the 20 SNPs significantly associated with ITCZ MIC when MIC was treated as a binary trait (Figure 2B), 12 SNPs were located in genes (11 in exons and one in an intron), two SNPs were located in 3′ UTR regions, 1 SNP was located in a 5′ UTR regions, and 4 SNPs were located in intergenic regions (Table 1). Of the 11 SNPs located in exons, six were synonymous (in *Afu2g02220, Afu2g02140, Afu2g02290, Afu2g02170*, and *Afu2g01910*) while the remaining five were non-synonymous (in *Afu2g01930, Afu2g02140, and Afu2g01910*) (Table 1). Interestingly, in this analysis, 19 of the 20 SNPs with lowest *p-values* were located to a 165 KB region on chromosome 2 (position 413,387 – 579,284) (Figure 2B).

Two significant SNPs overlapped between the quantitative trait and binary trait GWA analyses (Figure 2C). The SNP located in *Afu2g02220* encodes a synonymous variant and had the ninth lowest and lowest *p*-values in the quantitative trait and binary trait analyses, respectively (Figures 2A,B). *Afu2g02220* is annotated as a sterol 3-β-glucosyltransferase (Table 1). The SNP located in *Afu2g02140* encodes a non-synonymous variant (A233G) and had the 10th lowest and seventh lowest *p-*values in the quantitative trait and binary trait analyses, respectively (Figures 2A,B). *Afu2g02140* contains a CUE domain (as predicted by PFAM) (El-Gebali et al., 2019), which has been shown to bind to ubiquitin (Donaldson et al., 2003; Shih et al., 2003). For both *Afu2g02220* and *Afu2g02140*, the major allele was associated with higher MIC values and the minor allele was absent in all isolates with ITCZ MIC = 1, and nearly absent in isolates with ITCZ MIC = 0.5 (Figure 2D, Supplementary Figure 4).

### Expression of *Afu2g02220* and *Afu2g02140* From Existing RNA-Seq Experiments

To investigate whether gene expression of *Afu2g02220* and *Afu2g02140* could be modulated by environmental stress, we analyzed *A. fumigatus* RNA-seq data publicly available on FungiDB (Stajich et al., 2012), during oxidative stress, iron depletion, ITCZ exposure, and growth in blood and minimal media (Irmer et al., 2015; Kurucz et al., 2018). *Afu2g02220* was up-regulated during iron starvation (FPKM_control_ = 20.33, FPKM_FeStarvation_ = 32.70, and *p*-value = 5.7e^−4^), oxidative stress induced by H_2_O_2_ (FPKM_control_ = 20.33, FPKM_H2O2_ = 30.61, and *p*-value = 2.7e^−3^), iron starvation + H_2_O_2_ (FPKM_control_ = 20.33, FPKM_FeStarvation+H2O2_ = 39.93, and *p*-value = 6.7e^−23^), and during exposure to ITCZ in strain A1160 (FPKM_−ITCZ_ = 48.20, FPKM_+ITCZ_ = 67.37, and *p*-value = 1.7e^−4^) (Supplementary Figures 5A,B). *Afu2g02140* was not significantly up-regulated during any condition, and expressed at lower levels across all conditions compared to *Afu2g02220* (Supplementary Figure 5).

### Validation of a GWA Candidate Gene *via* CRISPR/Cas9 Gene Deletion

**Figure 3:**
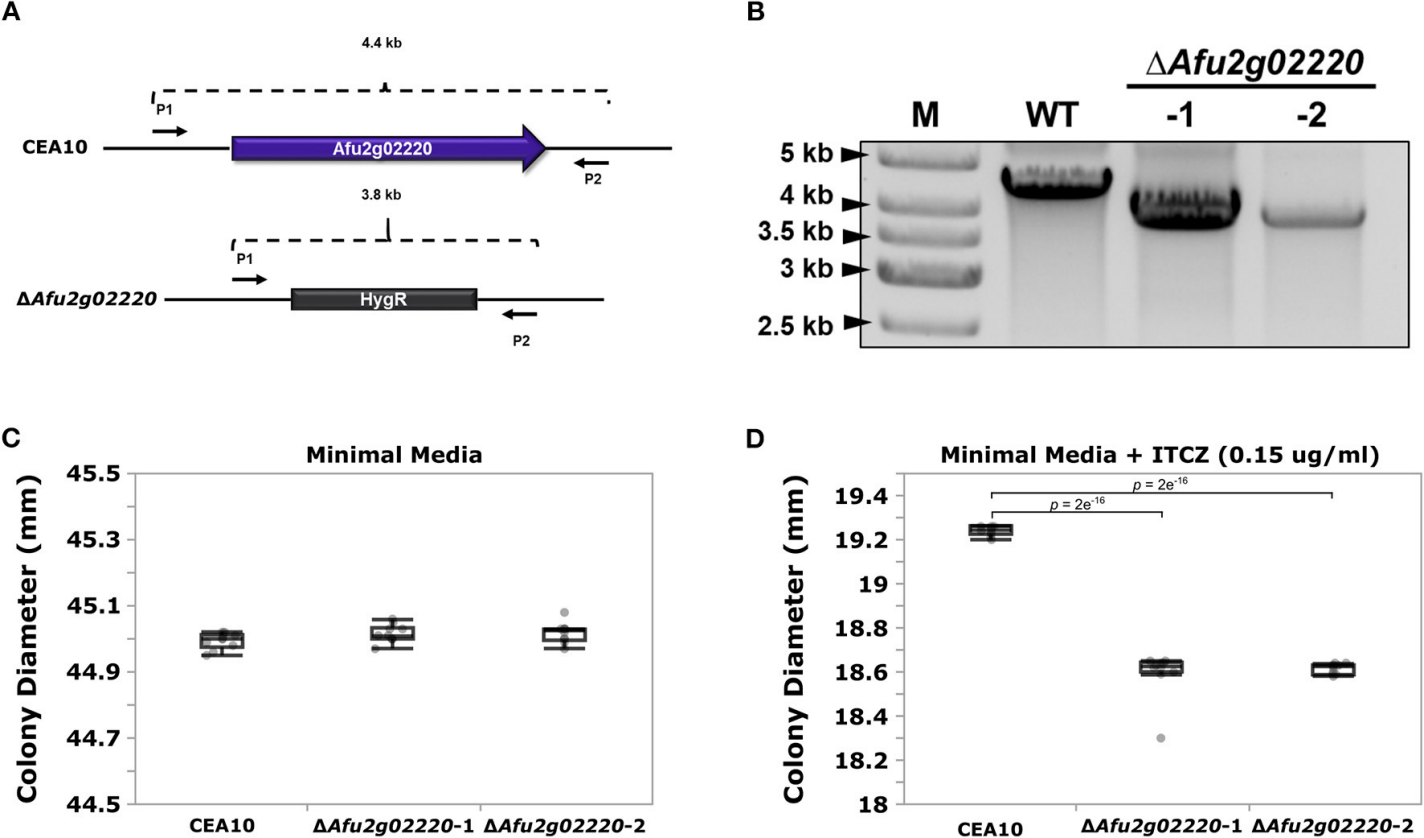
Deletion of *Afu2g02220* impairs growth in the presence of itraconazole (ITCZ). **(A)** Schematic of *Afu2g02220* gene deletion *via* CRISPR/Cas9. The blue box with arrow in the upper panel represents *Afu2g02220* in the parent CEA10 genome (wild type, WT), while the gray box in the lower panel represents the indicator gene *HygR* that replaced *Afu2g02220* in Δ*Afu2g02220* strains. The two black arrows on the flanking region of the locus indicate the forward primer (P1) and reverse primer (P2) used for PCR validation. The WT amplicon size is ~4.4 Kb, while the *HygR* gene replacement amplicon is ~3.8 Kb. **(B)** Validation of *Afu2g02220* gene replacement *via* PCR. Lanes “M,” “WT,” “-1,” and “-2” indicate ladder, PCR product from WT and PCR product from the two independent knockout strains, respectively. Boxplots for colony diameter of WT and Δ*Afu2g02220* strains grown at 37°C for 72 h on minimal media **(C)** and minimal media with 0.15 μg/ml ITCZ **(D)**. Measurements were collected for 10 biological replicates for each experiment. Dunnett's test *p-*values indicate a significant reduction in growth in the KOs compared to the WT.

We chose to functionally examine the role of *Afu2g02220* because (i) the SNP located in this gene had highly significant *p*-values in both GWA analyses (ii) *Afu2g02220* has a predicted functional role in sterol metabolism, and ITCZ targets the ergosterol biosynthesis pathway and (iii) *Afu2g02220* was up-regulated during ITCZ exposure (Supplementary Figure 5). Thus, we used an established CRISPR/Cas9 method (Al Abdallah et al., 2017) to knockout (KO) *Afu2g02220* by replacing it with the indicator gene hygromycin B phosphotransferase *(hygR)* in the *A. fumigatus* CEA10 genetic background (Figure 3A). We generated two independent KOs of *Afu2g02220* which we validated by *via* PCR (Figure 3B).

To test the effect of *Afu2g02220* on ITCZ sensitivity, we grew the wild type (WT) and Δ*Afu2g02220* strains in the presence of 0.15 μg/ml of ITCZ and measured colony diameter after 72 h of incubation at 37°C. We observed a qualitative reduction in conidia production in KO strains (Supplementary Figure 6). In minimal media without ITCZ Δ*Afu2g02220-1* and Δ*Afu2g02220-2* growth rates did not significantly differ from the WT (Δ*Afu2g02220-1* = 45.016 ± 0.027 mm, Δ*Afu2g02220-2* = 45.018 ± 0.030 mm, WT = 44.994 ± 0.024 mm) (Figure 3C). This result suggests that the background growth rate of Δ*Afu2g02220* is not impacted by the gene deletion. However, at ITCZ concentrations of 0.15 μg/ml we observed a minor but consistent reduction in growth in KO strains compared to WT (Δ*Afu2g02220-1* = 18.594 ± 0.105 mm, Δ*Afu2g02220-1* = 18.615 ± 0.022 mm, WT = 19.239 ± 0.021 mm) (*p*-value = 2e^−16^ for both KOs) (Figure 3D). These results suggest that *Afu2g02220* plays a minor role in ITCZ sensitivity.

## Discussion

Here, we analyzed the association between SNP allele frequency and ITCZ MIC data from 76 Japanese clinical isolates of *A. fumigatus* to identify loci involved in ITCZ sensitivity. MIC values fell within a relatively tight range of 0.125–1 μg/ml [for reference, ITCZ resistant strains are defined by MICs ≥ 4 μg/ml (Tashiro et al., 2012)]. We reasoned that GWA could be a feasible tool to identify loci that contribute to the small differences in ITCZ MIC we observed across these clinical isolates. We identified several candidate SNPs and loci associated with ITCZ sensitivity, and validated the function of the top candidate by knocking it out using a CRISPR/Cas9 based approach.

We identified a synonymous variant in *Afu2g02220* that showed highly significant associations with ITCZ sensitivity across GWA analyses with different underlying statistical models (Figure 2). Synonymous mutations can be functional through their (i) effect on cis-regulatory regions (e.g., splice sites or miRNA and exonic transcription factor binding sites), (ii) alteration of mRNA structure, or (iii) influence on translation speed (e.g., codon usage) (Hunt et al., 2014). Determining the mechanism by which this variant alters phenotype would require extensive *in silico* and *in vitro* experimentation. *Afu2g02220* encodes a predicted sterol glycosyltransferase. This enzyme biosynthesizes sterol glucosides, which make up the common eukaryotic membrane bound lipids. Orthologs of *Afu2g02220* from the ascomycete yeasts *Saccharomyces cerevisiae (Atg26), Candida albicans, Pichia pastoris*, as well as the amoeba *Dictyostelium discoideum* can use various sterols, including ergosterol, as sugar acceptors (Warnecke et al., 1999). In *S. cerevisiae*, Atg26 can directly bind to and glycosylate ergosterol, which yields ergosterol-glucoside (Gallego et al., 2010). In *S. cerevisiae* Δ*Atg26* did not impair growth when cultured in complex or minimal media, low or elevated temperatures, varying osmotic stress conditions, or in the presence of nystatin, an antifungal drug that binds to ergosterol (Warnecke et al., 1999). Similarly, we did not observe a difference in growth rate between Δ*Afu2g02220* and the WT when grown in minimal media (Figure 3C).

In addition to its role in sterol modification, *Afu2g02220* may also have additional functions related to autophagy (Kikuma et al., 2017). Orthologs of *Afu2g02220* in *Pichia pastoris* (*PpAtg26*) (Oku et al., 2003), *Colletotrichum orbiculare* (*CoAtg26*) (Asakura et al., 2009) and *Aspergillus oryzae* (*AoAtg26*) (Kikuma et al., 2017) are required for autophagy. In *A. oryzae*, Δ*AoAtg26* shows deficiency in degradation of peroxisomes, mitochondria, and nuclei and localizes to vacuoles (Kikuma et al., 2017). Δ*AoAtg26* also shows reductions in conidiation and impairment of aerial hyphae formation (Kikuma et al., 2017). Similarly, we observed a reduction in condition in Δ*Afu2g02220* compared to the WT (Supplementary Figure 6).

The fungal cell wall is rigid but also dynamic in order to respond to environmental stress. Because Afu2g02220 may directly interact with ergosterol, we hypothesized that environmental stress could alter the expression of *Afu2g02220*. We analyzed *A. fumigatus* RNA-seq data during growth under iron depletion, oxidative stress, ITCZ exposure and growth in blood and minimal media (Stajich et al., 2012). We found that *Afu2g02220* expression was significantly up-regulated during oxidative stress, iron depletion and ITCZ exposure (Supplementary Figure 5). However, other studies examining gene expression (da Silva Ferreira et al., 2006; Hokken et al., 2019) or protein abundance (Amarsaikhan et al., 2017) during exposure to ITCZ and voriconazole (da Silva Ferreira et al., 2006) (another triazole with the same mechanism of action as ITCZ) did not observe differential abundance of the Afu2g02220 transcript or protein. Additional experiments are necessary to determine the precise role of *Afu2g02220* in stress response and ITCZ sensitivity.

Previously, Palma-Guerrero et al. (2013) used a similar approach to identify NCU04379 as a gene that contributes to fungal communication in *N. crassa*. This study used RNA-seq data to identify genetic variants, Fisher's exact tests to perform GWA in a closely related group of 112 isolates, and existing deletion mutants generated by the *Neurospora* Genome Project (Colot et al., 2006; Dunlap et al., 2007) to validate the involvement of NCU04379 in cellular communication during germling fusion. A study in *S. cerevisiae* used a mixed linear model to identify correlations between genotype and tolerance to hydrolysate toxins, and used homologous recombination to knockout candidate genes in two independent genetic backgrounds (Sardi et al., 2018). Interestingly, eight of 14 gene knockouts had a significant effect on phenotype in one, but not both genetic backgrounds, suggesting that the network of genes contributing to hydrolysate toxins tolerance likely differs between genetic backgrounds. The results of these studies, and of our own, broadly suggest that GWA in combination with an efficient gene disruption technique is a powerful and unbiased approach for identifying the genetic basis of polygenic phenotypes in fungal systems.

## Data Availability Statement

Raw whole-genome Illumina data for the 65 isolates are available through NCBI BioProject PRJNA638646 and the 11 previously sequenced isolates by Takahashi-Nakaguchi et al. (2015) through NCBI BioProject PRJDB1541.

## Author Contributions

SZ and JG designed the study and analyzed the data. AW determined itraconazole MIC and provided *A. fumigatus* isolates. WG and JF conducted CRISPR and growth rate experiments. All authors contributed to writing the manuscript.

## Conflict of Interest

The authors declare that the research was conducted in the absence of any commercial or financial relationships that could be construed as a potential conflict of interest.
