# Supplementary material for: Genome-Wide Association for Itraconazole Sensitivity in Non-resistant Clinical Isolates of Aspergillus fumigatus
Source: Front Fungal Biol. 2021 Jan 14;1:617338. doi: 10.3389/ffunb.2020.617338 (PMC10512406; doi:10.3389/ffunb.2020.617338)
Supplement: Supplementary file 1 [file Image_1.pdf]

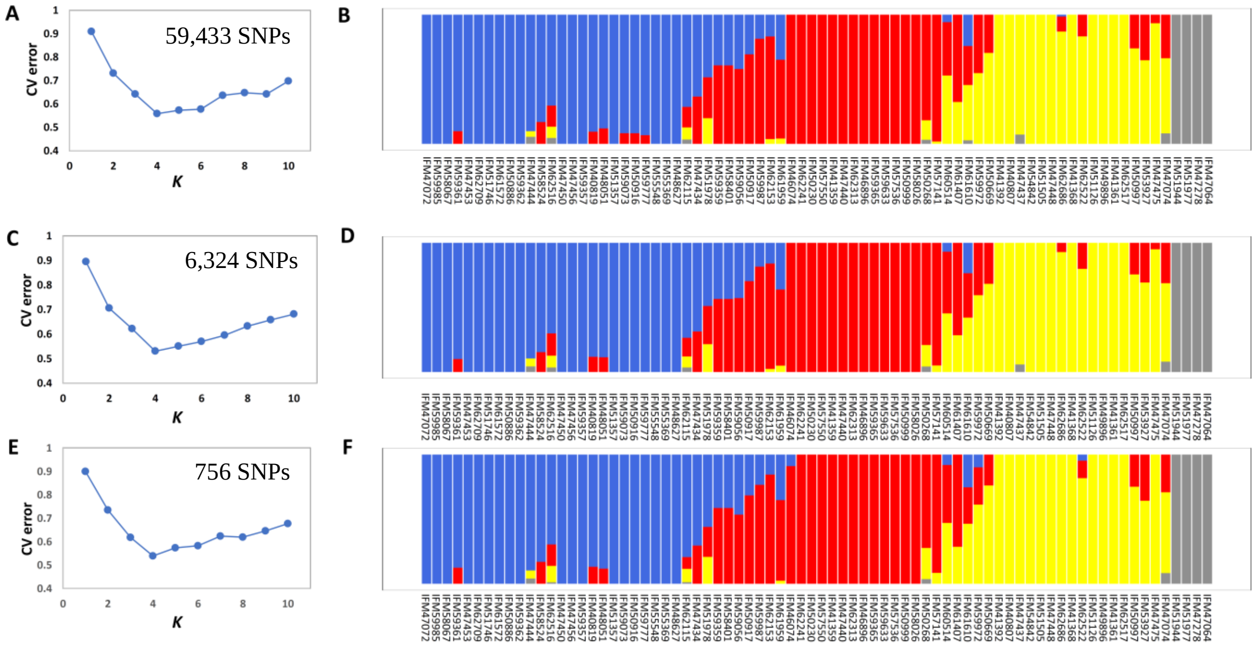

Figure S1. ADMIXTURE based population structure is consistent between SNP marker sets. The optimal predicted population number (*K*) (X-axis) estimated with the CV error (Y-axis) (A) and membership coefficient plots of the 76 isolates (X-axis) from ADMIXTURE for *K*=4 (B) using 59,433 SNPs. The CV error plot (C) and membership coefficient plots for *K*=4 (D) using 6,324 SNPs (with at least 3.5 kb distance between SNPs). The CV error plot (E) and membership coefficient plots for *K*=4 (F) using 756 SNPs (with at least 35 kb distance between SNPs). Population 1, 2, 3 and 4 are colored as blue, red, yellow, and gray, respectively.
