# Supplementary material for: Genome-Wide Association for Itraconazole Sensitivity in Non-resistant Clinical Isolates of Aspergillus fumigatus
Source: Front Fungal Biol. 2021 Jan 14;1:617338. doi: 10.3389/ffunb.2020.617338 (PMC10512406; doi:10.3389/ffunb.2020.617338)
Supplement: Supplementary file 2 [file Image_2.pdf]

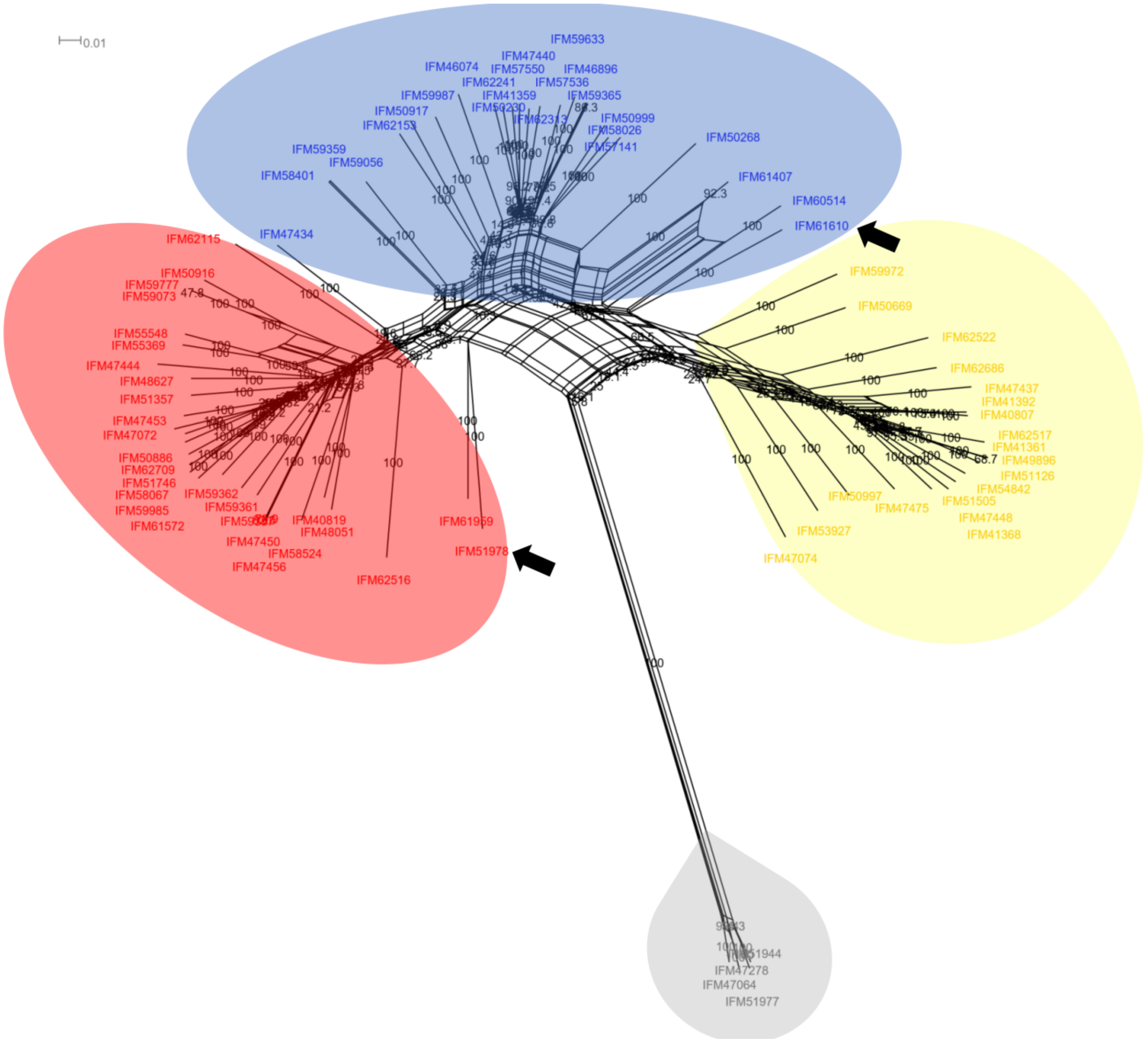

Figure S2. Phylogenetic network of the 76 *A. fumigatus* Japanese clinical isolates. The scale bar represents the proportion of nucleotide differences between two isolates. Isolates that are assigned to the DAPC based populations 1, 2, 3 and 4 are colored as blue, red, yellow, and gray, respectively. The two isolates that are assigned into different populations by ADMIXTURE and DAPC are indicated with black arrows. Bootstrap values are indicated.
