# Supplementary material for: Genome-Wide Association for Itraconazole Sensitivity in Non-resistant Clinical Isolates of Aspergillus fumigatus
Source: Front Fungal Biol. 2021 Jan 14;1:617338. doi: 10.3389/ffunb.2020.617338 (PMC10512406; doi:10.3389/ffunb.2020.617338)
Supplement: Supplementary file 3 [file Image_3.pdf]

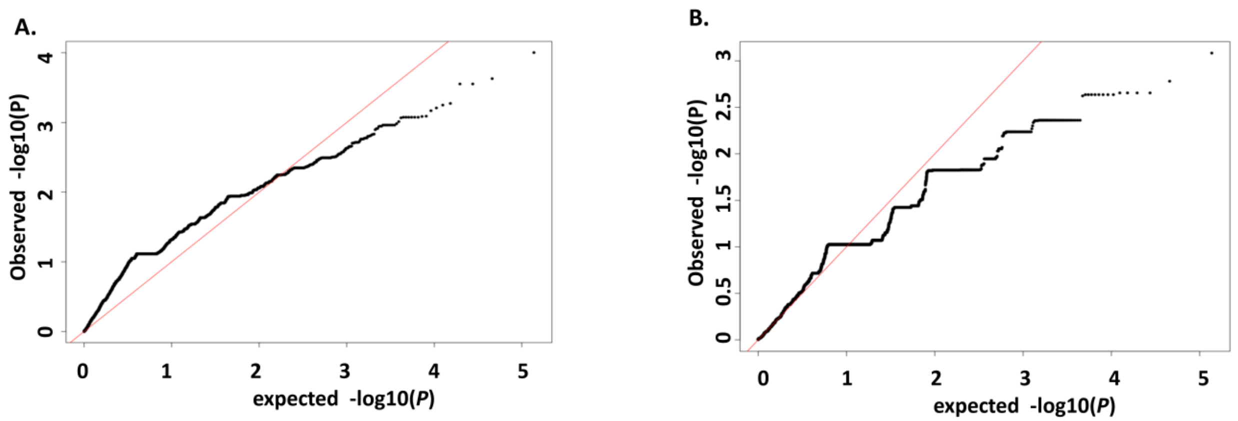

Figure S3. Quantile-quantile (Q-Q) plots of −log10 (*P-value*) from GWA analysis of *A. fumigatus* ITCZ sensitivity using Tassel (A) and RoadTrips (B). In each of the plots, Y-axis displays the quantile distribution of observed −log10 (*P-value*) while X-axis shows the quantile distribution of expected -log10 (*P-value*).
