# Supplementary material for: Genome-Wide Association for Itraconazole Sensitivity in Non-resistant Clinical Isolates of Aspergillus fumigatus
Source: Front Fungal Biol. 2021 Jan 14;1:617338. doi: 10.3389/ffunb.2020.617338 (PMC10512406; doi:10.3389/ffunb.2020.617338)
Supplement: Supplementary file 4 [file Image_4.pdf]

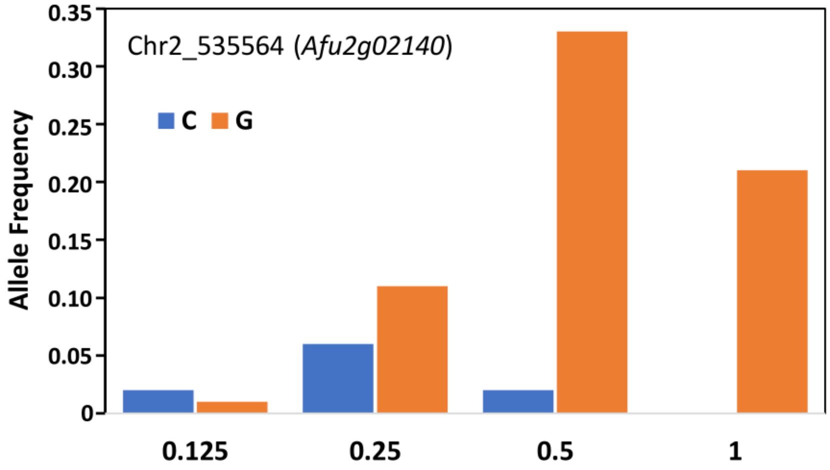

Figure S4. Allele frequency of the SNP in *Afu2g02140* that is associated with ITCZ sensitivity. The allele frequency of each allele is displayed on the Y-axis across different ITCZ MICs (X-axis). Blue and orange bars represent the minor and major alleles, respectively.
