# Supplementary material for: Genome-Wide Association for Itraconazole Sensitivity in Non-resistant Clinical Isolates of Aspergillus fumigatus
Source: Front Fungal Biol. 2021 Jan 14;1:617338. doi: 10.3389/ffunb.2020.617338 (PMC10512406; doi:10.3389/ffunb.2020.617338)
Supplement: Supplementary file 5 [file Image_5.pdf]

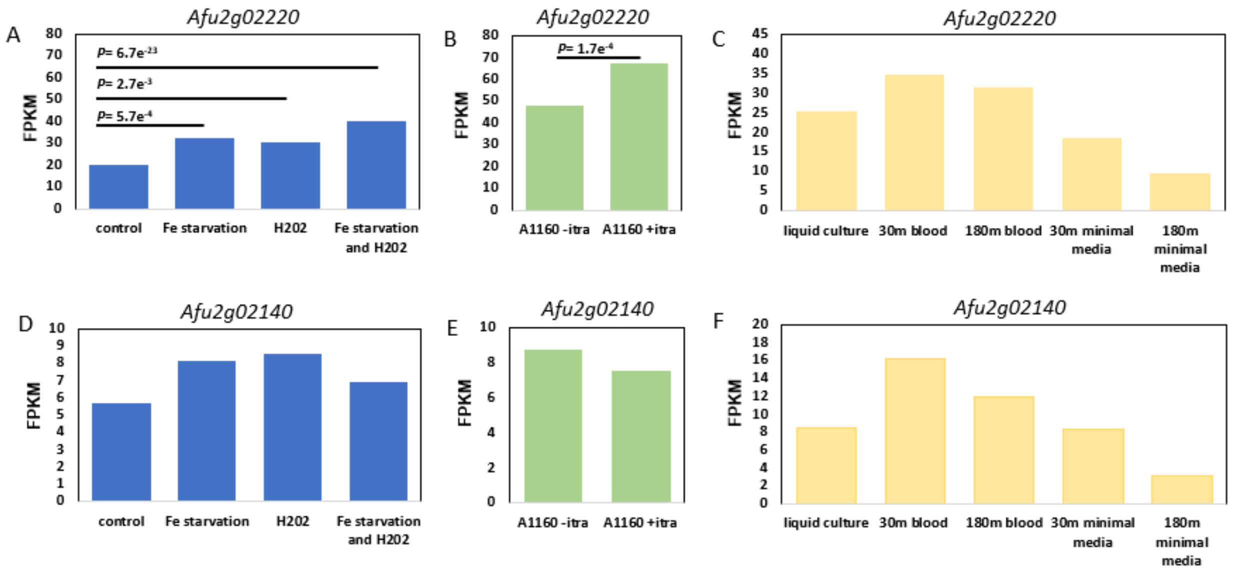

Figure S5. The expression of *Afu2g02220* (A, B, C) and *Afu2g02140* (D, E, F) in various conditions from RNA-seq data publicly available through FungiDB. FPKM (Fragments Per Kilobase of transcript per Million mapped reads) is displayed on Y-axis, while the X-axis represents experimental conditions. Bars are colored by study. *P*-values are reported for significant pairwise differential expression within the same study.
