# Supplementary material for: Genome-Wide Association for Itraconazole Sensitivity in Non-resistant Clinical Isolates of Aspergillus fumigatus
Source: Front Fungal Biol. 2021 Jan 14;1:617338. doi: 10.3389/ffunb.2020.617338 (PMC10512406; doi:10.3389/ffunb.2020.617338)
Supplement: Supplementary file 6 [file Image_6.pdf]

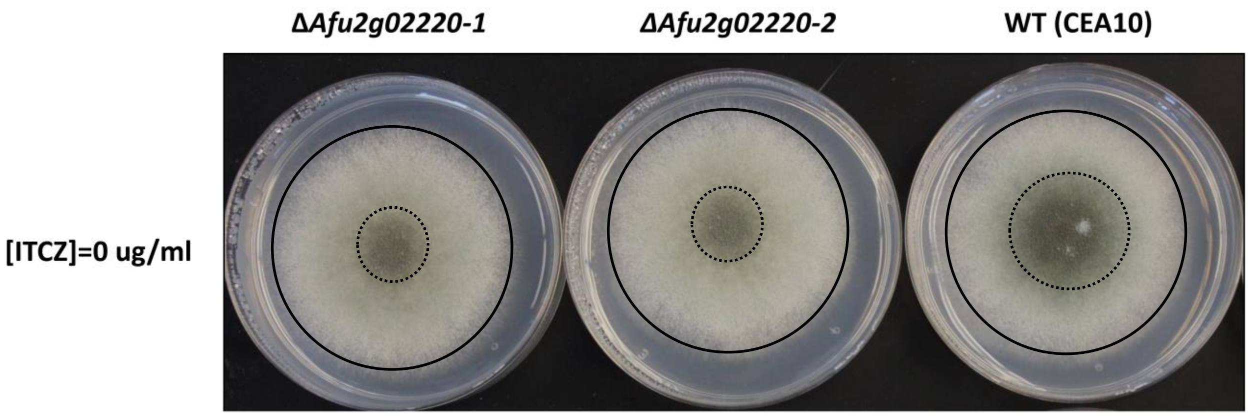

Figure S6. Growth of parent strain (CEA10) and *Afu2g02220* KOs on minimal media. The strains were incubated on General Minimal Media (GMM) without ITCZ at 37°C for 72 hours. The solid black circles are identical in size and indicate similarities in colony circumference. The dotted circles indicate the circumference of the sporulating region of the colony. KOs displayed a reduction in conidia production.
