# Supplementary material for: Genome-Wide Association for Itraconazole Sensitivity in Non-resistant Clinical Isolates of Aspergillus fumigatus
Source: Front Fungal Biol. 2021 Jan 14;1:617338. doi: 10.3389/ffunb.2020.617338 (PMC10512406; doi:10.3389/ffunb.2020.617338)
Supplement: Supplementary file 7 [file Table_1.DOCX]

**Table S1. Sample information for the 76 Japanese clinical *A. fumigatus* isolates.**

| **Sample ID** | **ITCZ MIC (ug/ml)** | **ITCZ MIC (binary)** | **Source** | **Place of isolation** | **Fungal disease of patient** | **BioProject Accession No.** | **BioSample Accession No.** |
| --- | --- | --- | --- | --- | --- | --- | --- |
| IFM40807 | 0.125 | 1 | Lung | Tokyo | CPA | PRJNA638646 | SAMN15199827 |
| IFM40819 | 0.5 | 2 | Lung | Tokyo | IPA | PRJNA638646 | SAMN15199828 |
| IFM41359 | 1 | 2 | Pleural effusion | Chiba | CPA | PRJNA638646 | SAMN15199829 |
| IFM41361 | 1 | 2 | Cornea | Chiba | Corneal ulcer | PRJNA638646 | SAMN15199830 |
| IFM41368 | 0.5 | 2 | Sinus | Tokyo | Sinusitis | PRJNA638646 | SAMN15199831 |
| IFM41392 | 0.5 | 2 | Sputum | Tokyo | CPA | PRJNA638646 | SAMN15199832 |
| IFM46074 | 0.5 | 2 | Lung | Chiba | CPA | PRJNA638646 | SAMN15199833 |
| IFM46896 | 0.25 | 1 | Sputum | Chiba | CPA | PRJNA638646 | SAMN15199834 |
| IFM47064 | 0.25 | 1 | Sputum | Chiba | CPA | PRJNA638646 | SAMN15199835 |
| IFM47072 | 0.25 | 1 | Sputum | Chiba | CPA | PRJNA638646 | SAMN15199836 |
| IFM47074 | 0.125 | 1 | Sputum | Chiba | CPA | PRJNA638646 | SAMN15199837 |
| IFM47278 | 0.25 | 1 | Sputum | Chiba | CPA | PRJNA638646 | SAMN15199838 |
| IFM47434 | 0.5 | 2 | Sputum | Chiba | CPA | PRJNA638646 | SAMN15486847 |
| IFM47437 | 0.25 | 1 | Sputum | Chiba | CPA | PRJNA638646 | SAMN15199839 |
| IFM47440 | 0.25 | 1 | bronchus | Chiba | CPA | PRJNA638646 | SAMN15199840 |
| IFM47444 | 0.5 | 2 | Sputum | Chiba | CPA | PRJNA638646 | SAMN15199841 |
| IFM47448 | 0.25 | 1 | Lung | Chiba | CPA | PRJNA638646 | SAMN15486848 |
| IFM47450 | 1 | 2 | Sputum | Chiba | ABPA | PRJNA638646 | SAMN15199842 |
| IFM47453 | 0.25 | 1 | Sputum | Chiba | CPA | PRJNA638646 | SAMN15199843 |
| IFM47456 | 0.125 | 1 | Lung | Chiba | CPA | PRJNA638646 | SAMN15199844 |
| IFM47475 | 0.5 | 2 | BALF | Chiba | CPA | PRJNA638646 | SAMN15199845 |
| IFM48051 | 0.25 | 1 | BALF | Tokyo | CPA | PRJNA638646 | SAMN15199846 |
| IFM48627 | 1 | 2 | Sputum | Chiba | CPA | PRJNA638646 | SAMN15486849 |
| IFM49896 | 1 | 2 | Sputum | Chiba | CPA | PRJNA638646 | SAMN15199847 |
| IFM50230 | 0.25 | 1 | Lung | Tokyo | CPA | PRJNA638646 | SAMN15199848 |
| IFM50268 | 0.25 | 1 | Sputum | Chiba | CPA | PRJNA638646 | SAMN15199849 |
| IFM50669 | 0.5 | 2 | unknown | Kyoto | Contamination | PRJNA638646 | SAMN15199850 |
| IFM50886 | 0.5 | 2 | Lung | Chiba | CPA | PRJNA638646 | SAMN15486850 |
| IFM50916 | 0.25 | 1 | BALF | Tokyo | CPA | PRJNA638646 | SAMN15199851 |
| IFM50917 | 1 | 2 | Sputum | Chiba | CPA | PRJNA638646 | SAMN15199852 |
| IFM50997 | 0.5 | 2 | Cutaneous aspiration | Kagoshima | Cutanous aspegillosis | PRJNA638646 | SAMN15199853 |
| IFM50999 | 0.5 | 2 | BALF | Chiba | CPA | PRJNA638646 | SAMN15199854 |
| IFM51126 | 0.5 | 2 | Sputum | Chiba | IPA | PRJNA638646 | SAMN15199855 |
| IFM51357 | 1 | 2 | Sputum | Tokyo | CPA | PRJNA638646 | SAMN15199856 |
| IFM51505 | 0.25 | 1 | Sputum | Chiba | CPA | PRJNA638646 | SAMN15199857 |
| IFM51746 | 1 | 2 | Sputum | Tokyo | CPA | PRJNA638646 | SAMN15199858 |
| IFM51944 | 0.5 | 2 | Sputum | Hokkaido | CPA | PRJNA638646 | SAMN15199859 |
| IFM51977 | 0.25 | 1 | Sputum | Chiba | CPA | PRJNA638646 | SAMN15199860 |
| IFM51978 | 0.5 | 2 | Sputum | Tokyo | CPA | PRJNA638646 | SAMN15199861 |
| IFM53927 | 0.5 | 2 | Sputum | Kochi | CPA | PRJNA638646 | SAMN15199862 |
| IFM54842 | 0.25 | 1 | Lung | Ishikawa | CPA | PRJNA638646 | SAMN15199863 |
| IFM55369 | 0.5 | 2 | Sputum | Chiba | IPA | PRJNA638646 | SAMN15199864 |
| IFM55548 | 0.25 | 1 | Cornea | Osaka | Corneal ulcer | PRJNA638646 | SAMN15486852 |
| IFM57141 | 0.5 | 2 | Sputum | Chiba | CPA | PRJNA638646 | SAMN15199865 |
| IFM57536 | 1 | 2 | Sputum | Chiba | Contamination | PRJNA638646 | SAMN15199866 |
| IFM57550 | 0.5 | 2 | Lung | Tottori | CPA | PRJNA638646 | SAMN15199867 |
| IFM58026 | 1 | 2 | Cornea | Fukuoka | Corneal ulcer | PRJDB1541 | SAMD00013550 |
| IFM58067 | 1 | 2 | Sputum | Ehime | CPA | PRJNA638646 | SAMN15199868 |
| IFM58401 | 0.5 | 2 | sputum |  |  | PRJDB1541 | SAMD00013548 |
| IFM58524 | 0.5 | 2 | Pleural effusion | Ibaraki | Empyema | PRJNA638646 | SAMN15199869 |
| IFM59056 | 1 | 2 | sputum |  |  | PRJDB1541 | SAMD00013552 |
| IFM59073 | 0.5 | 2 | Sputum | Chiba | CPA | PRJNA638646 | SAMN15199870 |
| IFM59357 | 1 | 2 | Sputum | Chiba | CPA | PRJNA638646 | SAMN15199871 |
| IFM59359 | 0.5 | 2 | Sputum | Chiba | CPA | PRJNA638646 | SAMN15199872 |
| IFM59361 | 1 | 2 | sputum |  |  | PRJDB1541 | SAMD00013551 |
| IFM59362 | 1 | 2 | Sputum | Chiba | Contamination | PRJNA638646 | SAMN15199873 |
| IFM59365 | 0.5 | 2 | sputum |  |  | PRJDB1541 | SAMD00013555 |
| IFM59633 | 0.5 | 2 | Sputum | Chiba | CPA | PRJNA638646 | SAMN15199874 |
| IFM59777 | 1 | 2 | Lung |  |  | PRJDB1541 | SAMD00013547 |
| IFM59972 | 0.5 | 2 | Lung | Osaka | CPA | PRJNA638646 | SAMN15199875 |
| IFM59985 | 1 | 2 | Sputum | Chiba | Contamination | PRJNA638646 | SAMN15199876 |
| IFM59987 | 0.5 | 2 | Sputum | Chiba | CPA | PRJNA638646 | SAMN15199877 |
| IFM60514 | 0.5 | 2 | sputum |  |  | PRJDB1541 | SAMD00013544 |
| IFM61407 | 0.25 | 1 | BALF |  |  | PRJDB1541 | SAMD00013549 |
| IFM61572 | 0.5 | 2 | Lung | Kumamoto | IPA | PRJNA638646 | SAMN15199878 |
| IFM61610 | 1 | 2 | BALF |  |  | PRJDB1541 | SAMD00013558 |
| IFM61959 | 0.5 | 2 | Pleural effusion | Aichi | CPA | PRJNA638646 | SAMN15199879 |
| IFM62115 | 0.5 | 2 | sputum |  |  | PRJDB1541 | SAMD00013556 |
| IFM62153 | 0.5 | 2 | Sputum | Chiba | CPA | PRJNA638646 | SAMN15199880 |
| IFM62241 | 0.5 | 2 | Sputum | Aichi | IPA | PRJNA638646 | SAMN15486855 |
| IFM62313 | 1 | 2 | Sputum | Chiba | CPA | PRJNA638646 | SAMN15199881 |
| IFM62516 | 1 | 2 | sputum |  |  | PRJDB1541 | SAMD00013559 |
| IFM62517 | 1 | 2 | Sputum | Tokyo | CPA | PRJNA638646 | SAMN15199882 |
| IFM62522 | 0.5 | 2 | Sputum | Tokyo | CPA | PRJNA638646 | SAMN15486856 |
| IFM62686 | 0.5 | 2 | Lung | Tokyo | CPA | PRJNA638646 | SAMN15199883 |
| IFM62709 | 0.5 | 2 | BALF | Kagoshima | CPA | PRJNA638646 | SAMN15199884 |

*Binary MIC 1 = ITCZ MIC < 0.5 ug/ml and 2 = ITCZ MIC ≥ 0.5 ug/ml

**ABPA = Allergic bronchopulmonary aspergillosis, CPA = Chronic pulmonary aspergillosis, IPA = Invasive pulmonary aspergillosis
